# Calcium-Dependent Protein Kinase 5 (*OsCPK5*) Overexpression in Upland Rice (*Oryza sativa* L.) under Water Deficit

**DOI:** 10.3390/plants12223826

**Published:** 2023-11-11

**Authors:** Thaís Ignez da Cruz, Dhiôvanna Corrêia Rocha, Anna Cristina Lanna, Beata Dedicova, Rosana Pereira Vianello, Claudio Brondani

**Affiliations:** 1 Escola de Agronomia, Universidade Federal de Goiás, Goiânia 74690-900, Brazil; thais_ignez@hotmail.com; 2 Instituto Agronômico de Campinas, Cordeirópolis 13490-970, Brazil; dhiovannarocha@gmail.com; 3 Embrapa Arroz e Feijão, Santo Antônio de Goiás 75375-000, Brazil; anna.lanna@embrapa.br (A.C.L.); rosana.vianello@embrapa.br (R.P.V.); 4 Department of Plant Breeding, Swedish University of Agricultural Sciences (SLU), Sundsvägen 10, P.O. Box 101, SE-230 53 Alnarp, Sweden; beata.dedicova@slu.se

**Keywords:** water-use efficiency, gene expression, senescence, drought

## Abstract

Water deficit significantly affects global crop growth and productivity, particularly in water-limited environments, such as upland rice cultivation, reducing grain yield. Plants activate various defense mechanisms during water deficit, involving numerous genes and complex metabolic pathways. Exploring homologous genes that are linked to enhanced drought tolerance through the use of genomic data from model organisms can aid in the functional validation of target species. We evaluated the upland rice *OsCPK5* gene, an *A. thaliana AtCPK6* homolog, by overexpressing it in the BRSMG Curinga cultivar. Transformants were assessed using a semi-automated phenotyping platform under two irrigation conditions: regular watering, and water deficit applied 79 days after seeding, lasting 14 days, followed by irrigation at 80% field capacity. The physiological data and leaf samples were collected at reproductive stages R3, R6, and R8. The genetically modified (GM) plants consistently exhibited higher *OsCPK5* gene expression levels across stages, peaking during grain filling, and displayed reduced stomatal conductance and photosynthetic rate and increased water-use efficiency compared to non-GM (NGM) plants under drought. The GM plants also exhibited a higher filled grain percentage under both irrigation conditions. Their drought susceptibility index was 0.9 times lower than that of NGM plants, and they maintained a higher chlorophyll a/b index, indicating sustained photosynthesis. The NGM plants under water deficit exhibited more leaf senescence, while the *OsCPK5*-overexpressing plants retained their green leaves. Overall, *OsCPK5* overexpression induced diverse drought tolerance mechanisms, indicating the potential for future development of more drought-tolerant rice cultivars.

## 1. Introduction

Water deficit is considered one of the principal environmental stresses for crops, and many regions of the world already face substantial water scarcity, with a consequent reduction in agricultural production [1]. Historically, most rice breeding efforts have been devoted to increasing yield potential, while currently, due to the problem of climate change, rice breeders are focusing on developing cultivars that are more resistant to abiotic stresses [2]. The initial plant physiological responses to water deficit include a decrease in leaf area, stimulation of leaf abscission, directional root growth towards moister soil regions, and induction of stomatal closure. In this context, the abscisic acid hormone (ABA) prevents excessive water loss during transpiration [3]. Stomata are essential plant gas exchange structures that regulate photosynthesis, respiration, transpiration, and temperature [4]. Furthermore, stomata are also the water-regulating organs of plants and are essential for water conservation, a decisive factor for the survival of plants under conditions of water deficit [5]. Water deficit can also induce a physiological imbalance due to an excess of reactive oxygen species (ROS), resulting in oxidative stress. In response, plants produce a variety of antioxidant enzymes, such as superoxide dismutase (SOD), peroxidase (POD), and antioxidant enzymes [6], to maintain their metabolic stability under situations of environmental stress.

Plant genes associated with water deficit tolerance encode proteins for cellular adaptation, including chaperones, transcription factors, or enzymes involved in signal transduction, such as protein kinases [7]. Numerous studies have been conducted in rice to elucidate the roles of different genes in water stress tolerance. Specific genes that have led to enhanced water deficit tolerance in rice include *OsEPF1* [8], *EDT1* [9], *OsRab7* [10], *OsMYB6* [11], *OsTF1L* [12], *OsbZIP42* [13], and *OsNAR2.1* [14]. Interestingly, several of these mentioned genes have significantly improved rice grain yield and plant biomass accumulation. However, the complexity of responses to water deficit suggests the involvement of a substantial number of genes that require further investigation for a more comprehensive understanding of stress tolerance mechanisms.

An increase in intracellular calcium concentration occurs in response to various stimuli, such as the accumulation of the hormone abscisic acid (ABA) in leaves during water deficit conditions [15]. Upon reaching the plasma membrane, ABA binds to membrane receptors, leading to an increase in cytosolic calcium that can occur either via the transient entry of Ca^2+^ ions into the intracellular environment or via the release of these ions from internal reservoirs, such as the endoplasmic reticulum and the vacuole. Such variation is essential in triggering signaling cascades in response to plant acclimatization. This process involves calcium-binding proteins that transmit the calcium signal, thereby inducing specific cellular and physiological responses [16].

Three main classes of Ca^2+^-binding proteins have been characterized in plants, namely calcium-dependent protein kinases (CDPKs), calmodulins, and B-like calcineurin proteins [17]. Among these classes, calcium-dependent protein kinases, commonly referred to as CPKs or CDPKs, are of particular significance due to their capacity to bind and transmit intracellular Ca^2+^ signals via a single gene product [18]. Such a combination may have originated in ancestral organisms through the fusion of protein kinase and calmodulin genes, wherein the former can modify other proteins by adding phosphate groups, while the latter can bind calcium [19]. Thus, CPKs can directly activate and regulate target proteins containing serine and threonine (Ser/Thr) residues upon detecting intracellular Ca^2+^ signals, facilitated via specific domains within their structural composition.

In plants, CPKs are widely distributed across various tissues, including the roots, stems, leaves, and flowers [20]. At the cellular level, they are abundant in the meristem, xylem, pollen, guard cells, and embryonic cells [21]. Subcellularly, they are found in the cytosol, nucleus, tonoplasts, mitochondria, chloroplasts, and peroxisomes, with the plasma membrane being the location where most CPKs are located in the model plant *Arabidopsis*. The diverse presence of CPKs in different parts of plants suggests their involvement in various signal transduction pathways [22]. It is well known that CPKs play multiple roles in plant biology, including senescence and cell death, hormone signal transduction, stress and defense responses, growth and development, carbon and nitrogen metabolism, cytoskeletal formation, and regulation of ion channels [23]. Protein kinases are species-specific and are encoded by a multi-gene family [24]. For instance, through genomic analysis, 34 CPK genes have been identified in *Arabidopsis* [25], and 31 CPK genes have been identified in rice [26].

Some CPKs have been functionally characterized in relation to water deficit tolerance in *Arabidopsis*. Transgenic plants overexpressing the *AtCPK6* gene have been found to exhibit increased tolerance to water and salt deficits, along with elevated transcription levels of this gene under stress conditions [27]. Several CPK genes appear to play a role in regulating stomatal opening and closing in *Arabidopsis*, a phenomenon also observed in maize with the *ZmCPK4* gene, which was found to increase water deficit tolerance by influencing stomatal closure through abscisic acid (ABA)-mediated pathways [28]. In *Arabidopsis*, the overexpression of *CPK10* was shown to lead to enhanced water deficit tolerance through the participation of *CPK10* in ABA- and calcium-mediated stomatal movements [29]. It is important to note that stomatal closure, despite reducing transpiration, limits gas exchange. This decrease in carbon dioxide assimilation generally results in reduced biomass and productivity in environments subjected to water deficits.

Functional studies involving different CPKs in relation to water deficit tolerance have already been conducted in rice. The *OsCPK4* gene has been associated with enhanced water retention capacity in plants overexpressing this gene [30]. Under water deficit conditions, the *OsCPK10* gene increased the hydrogen peroxide detoxification capacity in rice plants [31]. Overexpression of the *OsCPK9* gene led to improved stomatal closure and plant osmotic adjustment capacity, along with enhanced pollen viability and increased spikelet fertility [32]. The functions of CPK genes in rice, particularly in relation to water deficit tolerance, have not been yet fully understood, and studies involving gene overexpression in genetically modified plants (GMOs) compared to non-genetically modified plants can contribute to a better comprehension of the roles that these proteins play in plant adaptation to adverse water conditions. Such research also has the potential to aid in the development of commercial cultivars with increased drought tolerance. The objective of this work was to study the effect of the overexpression of *OsCPK5* in the genetically modified (GM) BRSMG Curinga upland rice plants in comparison to the non-genetically modified (NGM) BRSMG Curinga plants subjected to water deficit.

## 2. Results

The performance of the GM and NGM plants was compared in relation to two irrigation treatments. When comparing the harvest index (HI) of the GM and NGM plants, we observed that the GM plants subjected to the control treatment exhibited a HI 11.1% higher than that of the NGM plants in the same treatment (*p* < 0.05; Table 1). Conversely, no significant difference between the genotypes was observed in the water deficit treatment. Statistical differences (*p* < 0.05) were observed for each genotype (GM and NGM plants) across different irrigation treatments for the following traits: grain yield, number of filled grains, number of empty grains, and fresh mass (the latter only for the GM genotype). On the other hand, no significant difference was observed for the following traits: tiller number, panicle number, flag leaf length and width, and dry mass (Table 1).

The GM plants subjected to water deficit showed a drought susceptibility index (DSI) of 0.97 and were determined to be relatively tolerant to drought stress, according to the criteria established in [33]. Hence, the overexpression of the *OsCPK5* gene in GM plants was found to confer relative tolerance to water deficit, indicating that there would be a reduced drought impact on the performance of these GM upland rice plants. The DSI of 1.08 for BRSMG Curinga (NGM) indicates that this cultivar straddles a delicate balance between tolerance and sensitivity to water deficit, as observed by the authors of [34].

After 14 days of water deficit in the reproductive phase (stage R6), the transpiration rate (E) of GM plants was 42.1% lower than that of the NGM plants (*p* < 0.05; Table 2). At this stage, both the GM and NGM plants exposed to water deficit exhibited 44.5% and 23.2% lower transpiration rates than their corresponding control plants, respectively (*p* < 0.05). At the R6 stage, the decline in the transpiration rate (E) was the result of the reduction in stomatal conductance (gs), reaching 51.2 and 33.0% for the GM and NGM plants, respectively (Table 2). It is worth mentioning that the GM plants exhibited a stomatal conductance 46.7% lower than that of the NGM plants subjected to water deficit. Regarding carboxylation efficiency (A/Ci), no differences were evident between the GM and NGM genotypes under any irrigation treatment at any collection period. Moreover, it was observed that the GM plants maintained a consistent A/Ci across all collection periods in both irrigation treatments (Table 2).

Regarding the intrinsic water-use efficiency (iWUE), under the control treatment, the GM plants had an iWUE 40.7% higher than that of the NGM plants under the second collection period (R6 stage) (Table 2). Comparing the GM and NGM plants grown under the water deficit and control treatments at each data collection stage, it was observed that in the third collection period (7 days after the return of regular irrigation), the iWUE of the GM plants under water deficit was not significantly different from that of the corresponding plants in the control treatment. The NGM plants cultivated under water deficit had a 41.4% lower iWUE than the corresponding plants in the control treatment.

The GM plants also exhibited a notable intrinsic water-use efficiency (WUEintr). In the second collection period (stage R6), the GM plants under the control treatment displayed a WUEintr that was 40.2% higher than that of the NGM plants under the same irrigation conditions (Table 2). The higher WUEintr value in the GM plants occurred under the second collection period (stage R6) across irrigation treatments. Both genotypes showed higher WUEintr values 14 days after the irrigation cut-off relative to the collection periods. However, the GM plants were 40.2% more efficient in using water compared to their corresponding NGM plants under water deficit.

Discrepancies in the chlorophyll a and b proportions were observed between the GM and NGM genotypes during the first collection period (79 DAS, stage R3). In this period, the GM plants exhibited a chlorophyll a/b ratio that was 25% and 23.1% higher than that of the NGM plants under control and water deficit conditions, respectively (Table 3). In the second collection period (93 DAS, stage R6), no significant differences between the genotypes were observed in either irrigation treatment. However, during the third collection period (101 DAS, stage R8), the GM plants displayed chlorophyll a/b ratios that were 10.7% and 12% higher than those of the NGM plants under the control and water deficit treatments, respectively. No significant differences were observed in the chlorophyll a/b ratio during the first and second collection periods between plants of the same genotype grown under control conditions and those grown under water deficit treatment. However, during the third collection period, NGM plants exhibited a notable difference of 13.7% in the chlorophyll a/b ratio between plants grown under the control and water deficit treatments.

**Figure 1:**
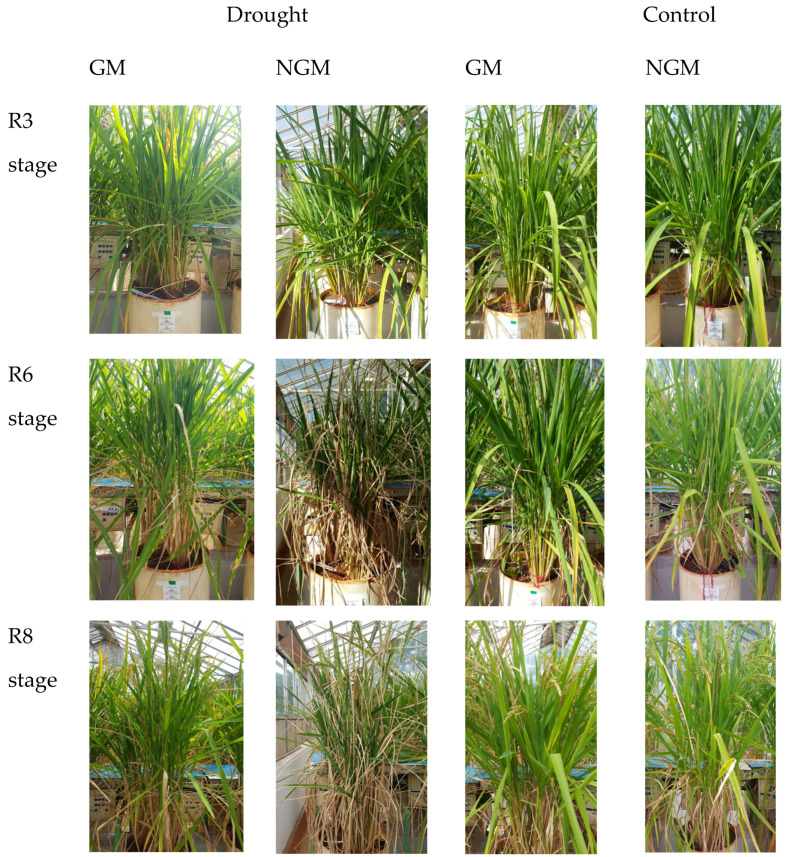
Phenotypic comparison between the GM and NGM plants conducted across three developmental stages.

Through visual comparisons of the general appearance of the GM and NGM plants subjected to water deficit (Figure 1), it was observed that in the second collection period (93 DAS, stage R6), 14 days after irrigation cut-off, the GM plants sustained a green coloration of their leaves. Conversely, the NGM plants exhibited numerous senescent leaves from the second collection period, a condition that persisted into the third collection period (101 DAS, stage R8), even following the restoration of irrigation.

**Figure 2:**
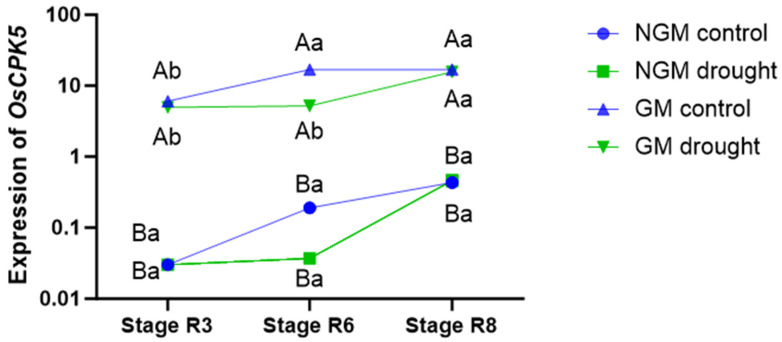
*OsCPK5* gene expression levels of NGM (wild-type) and GM (overexpressing *OsCPK5*) plants. The lowercase letter indicates significant differences in the genotype over the collection periods (stages R3, R6, and R8); the capital letter indicates differences between the NGM and GM genotypes under each collection period (Tukey’s test, *p* < 0.05, and n = 4).

The GM plants exhibited higher *OsCPK5* gene expression levels compared to the NGM plants under all collection periods and in both irrigation treatments (Figure 2). In the control treatment, the GM plants showed the highest level of *OsCPK5* gene expression during period 2 (stage R6), and this pattern persisted in period 3 (stage R8). For the GM plants subjected to the water deficit treatment, the highest expression of the *OsCPK5* gene was observed in period 3 (stage R8), 7 days after the return of regular irrigation. No significant difference in *OsCPK5* gene expression was observed between NGM plants under the control irrigation treatment and those under the water deficit treatment under any of the collection periods. Furthermore, in the NGM plants, there was no significant difference in the expression of the *OsCPK5* gene among the three collection periods under either irrigation treatment.

**Figure 3:**
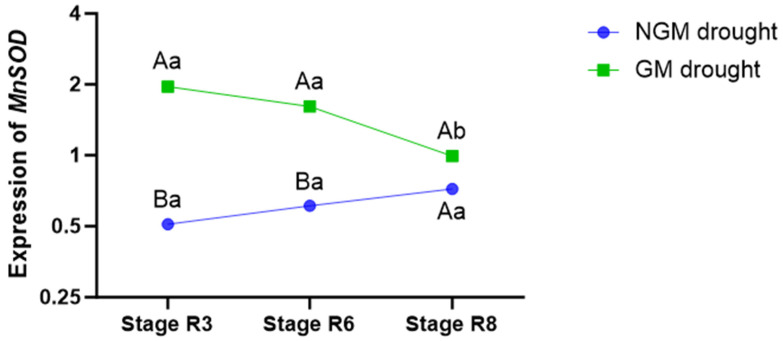
Gene expression of the *MnSOD* gene in NGM (wild-type) and GM plants (overexpressing *OsCPK5*). Lowercase letters denote significant differences within each genotype across the collection periods (stages R3, R6, and R8), while capital letters indicate disparities between the NGM and GM genotypes for each collection period (Tukey’s test, *p* < 0.05, and n = 4).

In order to investigate the underlying factors contributing to the delayed leaf senescence in the GM plants, we examined the gene expression of *MnSOD*, an essential component in the defense against oxidative stress in rice plants subjected to water deficit conditions. Compared to the NGM plants, the GM plants exhibited higher transcription levels of this gene starting from the first collection period (stage R3), and this elevated expression persisted (*p* < 0.05) until the end of the water deficit phase, corresponding to the second collection period (stage R6). Seven days after the return of irrigation (stage R8), the *MnSOD* expression level of the GM plants cultivated under water deficit conditions equaled that of the NGM plants under the same irrigation treatment (Figure 3).

RNA-seq generated an average of 43.6 Gbp of clean reads, of which 41.7 Gb (95.6%) were aligned to the rice reference genome (Table 4). Five billion reads were aligned to exons (89.3%), with the highest alignment achieved in well-annotated reference genomes. The 10 most expressed genes, determined via the abundance of transcripts mapped to the genome, are described in Table 5. For illustrative purposes, the transcript of the *OsCPK5* gene used in the transformation (Os02g0685900) has been added to Table 5.

Gene expression level analysis stands as a central task in a RNA-seq experiment, with the gene expression level being computed through the count of mapped reads. When contrasting the libraries of the GM and NGM plants exposed to drought conditions, the FPKM count for the Os02g0685900 transcript (*OsCPK5*) reached 233,193,538,410,951 (upregulated) for the GM plants, whereas for the NGM plants, it amounted to only 0.52.

A functional classification of GM vs. NGM sequences from drought libraries based on a gene ontology (GO) analysis revealed two groups of significantly upregulated genes: those related to biological process (BP), encompassing 13 classes, with “response to the oxygen-containing compound” showing the highest gene count at 22, and those related to molecular function (MF), representing four categories, with the category “unfolded protein binding” hosting 10 genes. Regarding significantly downregulated genes, three GO groups were identified: BP, with 89 classes, where “defense response” featured 43 genes; MF comprising 19 classes, with “carbohydrate binding” harboring 34 genes; and cellular component (CC) with one class, where “region extracellular” comprised 45 genes.

Examining the upregulated genes within the BP category, the classes most relevant to water deficit included “response to hydrogen peroxide” (GO:0042542), with six heat shock protein (hsp) genes, “response to reactive oxygen species” (GO:0000302) comprising five hsp genes, two peroxidases, and one aminotransferase, “osmotic stress response” (GO:0006970, see Table 6), and “oxidative stress response” (GO:0006979) with five hsp genes, two peroxidases, one aminotransferase, one HLH protein, and two unknown proteins). Notably, the last two classes were absent in the GO analysis of the GM × NGM control libraries. Among the downregulated genes in the BP category, the most directly related was “stress response” (GO:0080134, see Table 7). This class also did not appear in the GO analysis of the GM × NGM control libraries.

The enrichment analysis identified two statistically significant KEGG pathways associated with differentially expressed genes between the GM and NGM transcripts (the libraries from the drought treatment) (Table 8). None of these pathways were identified in the GM versus NGM libraries for the control treatment, but “Ribosome”, “Photosynthesis—antenna protein”, “Ribosome biogenesis in eukaryotes”, and “Phenylpropanoid biosynthesis” were found.

## 3. Discussion

This study highlighted how *OsCPK5* gene overexpression in GM plants impacts the regulation of stomatal closure, resulting in a decrease in stomatal conductance (gs) under WD conditions. Stomatal closure serves as the primary leaf response to drought conditions, minimizing water loss by decreasing the transpiration rate (E) and increasing the water-use efficiency (WUE) [35]. Notably, significant differences in gs and E were observed between the GM and NGM plants during the second collection period (stage R6), a critical phase when water loss intensifies due to grain filling in the panicles. Despite the identification of this important physiological difference between the two genotypes, with the GM plants being more efficient in their water use, both the GM and NGM plants presented the same grain yield. Certainly, this outcome arises from the intricate nature of grain productivity, influenced as it is via an array of metabolic pathways and genetic interactions.

To address water deficit, plants close their stomata to maintain cell turgor and metabolism, thereby affecting the photosynthetic rate [2]. Photosynthesis is highly sensitive to drought stress and is the foremost process that is altered by such conditions. Decreased production of photoassimilates reduces leaf growth and crop yield [36]. The enhancement of stomatal regulation, probably due to the overexpression of *OsCPK5*, has also been reported in studies involving other genes of the CPK family. For instance, the authors of [28] investigated the impact of overexpressing the corn *ZmCPK4* gene in *Arabidopsis thaliana* and found an amplified sensitivity of the plant to the hormone abscisic acid (ABA) that increased stomatal closure in response to water deficit. The authors of [32] observed that compared to control plants, transgenic rice plants overexpressing *OsCPK9* under normal growth conditions exhibited no significant differences in stomatal opening; however, following a period of water deficit, a higher proportion of fully closed stomata was observed in the transformed plants, in contrast to what was observed in the control plants, suggesting that the *OsCPK9* gene influences stomatal movement under water deficit conditions.

Water deficit-tolerant plants respond to drought mainly through fine control of stomatal and mesophilic conductance [37], and under these conditions impairment of CO_2_ assimilation may occur, as well as restrictions on growth and metabolism [38]. However, in this study, it was observed that the improvement in stomatal closure in the GM plants at stage R6 (the grain-filling phase), following a 14-day water deficit, did not lead to a reduction in the productivity of this genotype when compared to the NGM plants cultivated under similar irrigation treatments. Several factors may have contributed to decreasing the impact of water restriction on the productivity of GM plants relative to NGM plants, even after a substantial reduction in stomatal conductance and transpiration rate during the grain-filling phase. These factors include greater water-use efficiency during the grain-filling phase, delayed leaf senescence, and higher levels of chlorophyll a and b in the GM plants than in the NGM plants.

The stability in carboxylation efficiency observed in the GM plants under the three collection stages in both irrigation treatments could potentially be linked to enhanced protection against the degradation of Rubisco (ribulose-1,5-bisphosphate carboxylase/oxygenase). Another probable reason was that ribulose activase, the enzyme that regulates Rubisco activation [39], was the most expressed enzyme identified via RNA-seq, with the GM plants under water deficit having 9% more transcripts of this enzyme than the NGM plants under the same irrigation conditions and 18% more transcripts than the NGM plants in the control treatment. Furthermore, the elevated protection against the degradation of this enzyme after re-irrigation in the GM plants may have contributed to a better cellular redox balance, thereby creating a more favorable cellular environment for its preservation and activity. The carboxylation efficiency was also noted by the authors of [40] when evaluating the effect of water deficit on Kentucky bluegrass (*Poa pratensis* L.), a C3 perennial grass. In that study, the drought-tolerant genotype demonstrated an enhanced activity and state of activation of Rubisco following re-irrigation, restoring metabolic activity to levels comparable to those of control plants. This phenomenon was considered a plausible cause for the recovery of photosynthetic activity.

Water-use efficiency (WUE) is associated with the ability of plants to deal with varying degrees of water deficit and plays a pivotal role in maintaining productivity even when water availability is restricted [41]. During the critical water deficit period (R6 stage), the GM plants overexpressing the *OsCPK5* gene exhibited higher iWUE levels than the NGM plants. The iWUE level observed after the water deficit period suggested that the NGM plants were unable to restore their CO_2_ assimilation machinery, leading to malfunction of the photosynthetic apparatus and degradation of pigments, compromising the different stages of photosynthesis [42]. The correlation between gs reduction and greater water-use efficiency has been observed by the authors of [43,44], who evaluated C3 plants (such as rice) under moderate water deficit scenarios.

Reducing the gs per amount of CO_2_ assimilated and increasing the rate of CO_2_ assimilation can improve the WUEintr [45]. In the R6 stage, the GM plants overexpressing the *OsCPK5* gene showed higher WUEintr levels than the NGM plants under both irrigation treatments. In fact, in contrast to the NGM plants under water deficit, the GM plants exhibited a notable decrease in gs and E at 14 days after cutting off irrigation (stage R6), concomitant with an augmentation in WUEintr at this particular stage. In wheat, negative correlations among photosynthesis, transpiration, and stomatal conductance on water-use efficiency have been reported, suggesting that stomatal characteristics are factors responsible for regulating water-use efficiency in these plants [43]. Increasing the water-use efficiency of rice is critical, as rice grown under the lowland system require more than 2.5 kg of water for each grain of rice produced [2].

Visually, the GM rice plants overexpressing the *OsCPK5* gene showed a greener leaf appearance than the NGM plants during the grain-filling phase after 14 days of irrigation restriction, while the leaves of the NGM plants turned yellow and senescent. The correlation between the overexpression of a calcium-dependent protein kinase and the green appearance of leaves was also observed by the authors of [30] when examining the overexpression of *OsCPK4* to increase tolerance to water deficit in rice plants of the cultivar Nipponbare (*Oryza sativa* ssp. *japonica*). Visual differences between plants overexpressing the *OsCPK4* gene and control plants were observed from the 14th day without irrigation, when the control plants (wild type) showed symptoms of damage induced via water deficit, such as leaf wilting, which remained until the last day of cutting irrigation (17th day). On the other hand, transgenic plants overexpressing *OsCPK4* remained healthy and green. These authors verified that after rehydration of the plants, approximately 90% of the transgenic plants recovered from the stress, while none of the control plants survived the water deficit treatment. In our experiment, a similar result occurred, as the return of irrigation for 7 days (stage R8) failed to rejuvenate the NGM plants after the applied water deficit, increasing the senescent appearance of their leaves in comparison with those of the GM plants, where senescence was delayed. Plants with the stay-green trait are important in the case of drought, as the extension of the photosynthesis period leads to greater grain productivity [46].

According to the authors of [47], the relationship between the expression of CPK family genes and the senescence process in rice leaves was further underscored through the functional validation of the *OsCPK12* gene. In this context, they observed that mutant plants, in which the gene was knocked out, exhibited yellowing and senescent leaves during the grain-filling stage, while wild-type plants exhibited a greener appearance. Investigations into the possible reasons for this difference revealed that mutant plants had a greater accumulation of H_2_O_2_ and superoxide radicals in leaves, in addition to more cell death and apoptosis, than wild-type plants. On the other hand, *OsCPK12* overexpression led to higher photosynthetic rates, increased chlorophyll a and b contents, delayed leaf senescence, and a delayed plant growth period, providing positive effects for crop productivity.

There is a positive correlation between increased *SOD* gene expression and increased plant tolerance to environmental stresses [3]. In this work, the evaluation of the expression of *MnSOD*, important in preventing oxidative stress [48], revealed that the GM plants overexpressing *OsCPK5* grown under water deficit treatment presented a rapid and strong activation of this defense response from the R3 stage that persisted until the end of the irrigation cut during the reproductive phase. Levels of antioxidant metabolites and enzymes that regulate the cellular redox state increase under different stresses, i.e., a greater antioxidant activity confers greater tolerance to these stresses in plants [49]. Increased expression/activity of total superoxide dismutases (SODs) due to water deficit was observed in rice [50]. Furthermore, the authors of [51], when evaluating the enzymatic activity and gene expression levels of eight SOD isoforms in rice, observed that more SOD genes were expressed in the vegetative phase than under the reproductive phase. However, the enzymatic activity of SOD only increased during the reproductive phase of the plants.

In the current study, although quantification in the greenhouse experiment revealed that the values of the chlorophyll a/b ratio of the GM and NGM plants were not significantly different, the GM plants presented 35% more transcripts of the chlorophyll a/b binding protein-encoding gene than the NGM plants under water deficit, while the GM plants had 75% more transcripts than the NGM plants under control irrigated treatment. Light-harvesting chlorophyll a/b binding proteins, according to the authors of [52], play a role in the efficiency of photosynthesis, in addition to participating in the response to abiotic stresses. As pointed out by the authors of [3], an increased proportion of chlorophyll a and b is indicative of greater drought tolerance, which provides further evidence that the GM plants have a better response to drought. The relationship between overexpression of a calcium-dependent protein kinase and an increase in chlorophyll content was also observed by the authors of [32] following a water deficit treatment in rice plants overexpressing the *OsCPK9* gene (*Oryza sativa*, cultivar Nipponbare) in contrast to plants with silencing of this gene and control plants. The authors of [47], when investigating possible reasons for differences in chlorophyll content from overexpression or silencing of CPK family genes in *Oryza sativa* ssp. *indica*, noted that mutant plants of genes of the CPK family presented negative regulation of genes involved in the synthesis of chlorophylls and positive regulation of genes involved in the degradation of these pigments. This fact may indicate that overexpression of *OsCPK5* also acts on the synthesis or degradation pathways of these pigments in upland rice, paving the way for new investigations into which genes and metabolic pathways related to the synthesis or degradation of chlorophylls were altered from overexpression of the *OsCPK5* gene.

The leaves of the GM plants overexpressing the endogenous and exogenous *OsCPK5* gene showed higher transcription levels of this gene than the NGM plants under both irrigation treatments at the three evaluated collection periods (stages R3, R6, and R8). A similar result was obtained by the authors of [27] when examining the transcription levels of the *AtCPK6* gene, an orthologue of *OsCPK5*, in *A. thaliana*, in which the levels of the *AtCPK6* gene in transformed plants were higher than those in wild-type plants and associated with increased drought tolerance. The *OsCPK5* gene was poorly expressed in relation to the other transcripts identified via RNA-seq analysis. Even so, the transcription level of the *OsCPK5* gene was slightly higher in the GM plants than in the NGM plants, which indicates that the best alternative to monitor the presence of specific genes is quantitative PCR. The authors of [26], when evaluating CPK family genes in *Oryza sativa* ssp. *indica* using microarrays and RT-qPCR, observed that fourteen CPK genes were upregulated during the panicle development stage and six during the seed development stage, indicating that CPK expression is also regulated via the rice development stage.

The majority of the upregulated transcripts in the GM drought libraries identified via the GO analysis were “heat shock proteins” (HSPs), which have important roles in transducing cell signaling, regulating apoptosis, and protecting cells against biotic and abiotic stresses [53]. Conversely, the downregulated transcripts were mainly TIFY genes, which exert a regulatory function in plant development and responses to biotic and abiotic stresses [54], exhibiting a similar function to HSPs. The WRKY transcription factor, which plays a key role in transmitting and responding to drought stress signals, was also found to be downregulated [3]; that is, WRKY expression was higher in the NGM plants. According to the KEGG analysis, TIFY proteins were identified in the “plant hormone signal transduction” pathway, showing higher expression levels in the NGM plants than in the GM plants under water deficit. The other pathway identified via KEGG analysis was the “MAPK signalling pathway”, associated with the mitogen-activated protein kinase (MAPK) cascade, a defense mechanism induced against abiotic stresses in plants [55]. Most of the genes in this pathway were upregulated in the NGM plants, supporting the hypothesis that these plants are more adversely affected by the consequences of water deficit than GM plants.

## 4. Material and Methods

### 4.1. Gene Transformation

The *OsCPK5* gene (LOC_Os02g46090—TIGR ID; Os02g0685900—RAPDB ID), utilized in rice transformation, was initially identified from the sequence of its homologue in *Arabidopsis*, the *AtCPK6* gene (AT2G17290). The company DNA Cloning Service (Hamburg, Germany) carried out the cloning of the *OsCPK5* gene in the p7i2x-Ubi binary vector, which encompasses the 35S promoter for the bar gene, conferring tolerance to ammonium glufosinate and the ubiquitin promoter (Ubi-1) for the *OsCPK5* gene. Rice transformation for overexpression of the *OsCPK5* gene was described by the authors of [56] utilizing *Agrobacterium tumefaciens* (EHA 105 strain). The cultivar employed for the transformation was BRSMG Curinga, commercially released by Embrapa in 2005. The selection of the transformed calluses was conducted using the herbicides bialaphos and phosphinothricin (PPT), both of which contain ammonium glufosinate as an active ingredient, in culture medium. The calli that proliferated in the presence of this selection agent were sub-cultured, originating T0 plants that were subsequently subjected to PCR to amplify the bar gene to identify genetically modified (GM) plants. Then, the Southern blot technique was performed to estimate the number of copies inserted in the genome. Furthermore, to confirm the activity of the bar gene in the transformed rice, the chlorophenol red test was carried out using leaves from transgenic tillers of the T0 generation. The 17 independent genetic transformation events obtained were evaluated for water deficit tolerance and ideal plant type traits as they advanced through the generations (from T1 to T3). Additionally, the leaves were treated with a 2% ammonium glufosinate herbicide solution to select plants that were tolerant to this herbicide and, consequently, that carried the gene of interest. An experiment was carried out to evaluate the performance of three CPK events (2, 4, and 14) to identify the one with the best performance for drought tolerance (Supplementary Table S1). Ultimately, genetically modified (GM) event 4 (*OsCPK5*-E4, generation T4) was chosen for conducting the experiment both with and without water deficit to evaluate the physiological performance, productivity, and the expression profile of the plants with the *OsCPK5* and *MnSOD* (manganese superoxide dismutase) genes in comparison to the non-genetically modified (NGM) cultivar BRSMG Curinga.

### 4.2. Experimental Conditions

This experiment was conducted during the 2018/2019 season on a semi-automated phenotyping platform situated at the Embrapa Arroz e Feijão experimental station in Santo Antônio de Goiás City, Brazil (49°17′ W; 16°28′ S; 779 m altitude). The employed experimental design was a randomized block design, with four replicates for both the control and water deficit treatments. The plots consisted of PVC columns 40 cm in height and 30 cm in diameter filled with the dystrophic Red Clay Oxisol, a soil type typical of the Cerrado Biome, with its pH and fertility adjusted according to technical recommendations [57]. Within each column, two plants from event 4 were sown following the same procedure used for the NGM plants. The columns were placed on scales to determine when 80% of the soil’s field capacity was reached, as well as to monitor water loss and the necessary irrigation to maintain water content. Plants were irrigated once a day in the morning to reach the desired weight in each column. The irrigation treatments were: (1) irrigated (or control), with irrigation covering close to 80% of the soil’s field capacity; and (2) drought or water deficit, with restricted irrigation during the reproductive phase, starting 79 days after sowing and lasting 14 days. This limited irrigation approach aimed to maintain the column weight after each of the columns losing 4 kg of water. Over the 14-day period, the amount of water replaced in the columns subjected to water deficit was adjusted to 80% of the field capacity minus 4 kg. This replacement was necessary to induce a plant response to water deficit for a period of two weeks, and after this period irrigation returned to normal, as in the control treatment.

### 4.3. Physiological Measurements

The phenotypic performance of the GM plants overexpressing the *OsCPK5* gene in the T4 generation in comparison to that of the NGM BRSMG Curinga was assessed. The physiological data and plant material from both the GM and NGM plants were collected during three periods: period 1—beginning of the water deficit (79 days after sowing, at the panicle emission—reproductive stage R3, according the developmental system proposed by the authors of [58]; period 2—end of the water deficit (93 days after sowing, in the grain-filling phase—stage R6); and period 3—seven days after the return of irrigation to 80% of the soil field capacity (101 days after sowing, at the beginning of physiological maturation—stage R8). Physiological data were obtained from the middle segment of the flag leaf of the main stem of each plant between 8:00 am and 11:00 am across the three collection periods using a portable leaf chamber IRGA (infrared gas analyzer, model LCpro-SD, ADC BioScientific, Hertford, UK). The measured parameters were the photosynthetic rate (A) (μmol CO_2_ m^–2^ s^–1^), stomatal conductance (gs) (mol H_2_O m^–2^ s^–1^), transpiration rate (E) (mmol H_2_O m^–2^ s^–1^), and intracellular concentration of CO_2_ (Ci) (μmol mol^–1^). The employed photosynthetically active photon flux density (PFFD) was 1200 μmol [quanta] m^−2^ s^− 1^. The carboxylation efficiency was calculated as A/Ci for each plant; the water-use efficiency and instantaneous water-use efficiency (iWUE) (μmol CO_2_ mol^–1^ H_2_O) were calculated as iWUE=A/E; the intrinsic water-use efficiency (WUEintr) (μmol CO_2_ mol^–1^ H_2_O) was calculated as WUEintr = A/gs. The estimated chlorophyll a and b content was evaluated in the fully expanded flag leaves of each plant at each of the three collection periods using a portable chlorophyll meter ClorofiLOG^®^ model CFL 1030 (Falker Automação Agrícola, Porto Alegre, Brazil). The chlorophyll content values were employed to calculate the chlorophyll a and b ratio as Cl.a/Cl.b. These results were statistically analyzed using ANOVA and Tukey’s test (*p* < 0.05) in R software version 3.5.3.

Samples of healthy leaf tissue were collected at period 1 (stage R3), period 2 (stage R6), and period 3 (stage R8), seven days after the return of irrigation. These collected samples were promptly wrapped in aluminum foil, flash-frozen in liquid nitrogen, and preserved in an ultra freezer (−80 °C) for subsequent utilization in gene expression analysis via RT-qPCR. Thirty days after the beginning of flowering, the plants of the different treatments were ready for harvest. At that time, the length and width of three flag leaves from distinct tillers of each plant were measured, coinciding with the IRGA readings. A ruler was used to measure the length of the leaves, and a caliper was used to measure the width. Plant height (in centimeters) from the base of the stem to the apex of the highest panicle was determined. Upon harvest, the number of tillers and panicles of each plant was counted. The panicles were individually harvested for the purpose of weighing and quantifying the grains within each plant. The plants with removed panicles were trimmed near the soil surface, placed within labeled envelopes, and weighed to determine their fresh mass. Then, they were subjected to a drying process in an oven set at 60 °C for 72 h and were then weighed again to measure the dry mass.

In order to determine the yield data, the total weight of grains per plant was measured. Furthermore, the number of filled and empty grains of three random panicles from each plant was counted. Additionally, the drought susceptibility index (DSI) was determined according to the methodology outlined by the authors of [59], calculated through the formula:

$$\mathrm{ISS}=\frac{1-(\mathrm{Yd}/\mathrm{Yp})}{D}$$

where Yd is the yield value of the genotype under water deficit; Yp is the yield value of the genotype under normal irrigation; and D is the average yield (total average weight of grains per plant) of all the genotypes under water deficit conditions/average yield (total average weight of grains per plant) of all the genotypes under irrigation conditions. According to the authors of [33], the genotypes were classified as water deficit-tolerant when the DSI values were below 1, whereas genotypes with DSI values exceeding 1 were considered susceptible to water deficit.

The harvest index (HI), representing the grain yield relative to the total dry matter of the plant, reflects the physiological capacity of the plants to allocate photosynthates for grain filling in panicles. It was determined according to the formula proposed by the authors of [60]:

$$HI(\%)=\frac{grainyield}{biologicalyield}\times 100$$

where the grain yield signifies the grain weight per plant, while the biological yield accounts for the grain weight per plant plus dry mass weight, computed for each irrigation treatment. Statistical analysis was performed using ANOVA and Tukey’s test (*p* < 0.05) with R software version 3.5.3.

### 4.4. RNA Extraction and Real-Time PCR

Total RNA extraction was performed with the leaf tissue of four GM plants (two from the control treatment and two from the water deficit treatment), as well as four NGM plants. The leaf samples were collected during periods 1, 2, and 3. The extractions were carried out using the RNeasy Plant Mini kit (Qiagen, Germantown, MD, USA) according to the manufacturer’s instructions, followed by DNase treatment of total RNA (RNase Free DNA, Qiagen). The extracted RNA was resuspended in 50 µL of RNase-free water and kept at −80 °C.

The RNA samples were evaluated in a Qubit^®^ 2.0 Fluorometer (Thermo Fisher, Waltham, MA, USA) to quantify total RNA. The integrity of the RNA molecules was evaluated using the Agilent RNA 6000 Nano kit on the Bioanalyzer 2100 device (Agilent Technologies, Santa Clara, CA, USA). A reverse transcription reaction (RT) was conducted to generate cDNA molecules from 1 µg of RNA using the GoScript reverse transcription system kit (Promega, Madison, WI, USA), as per the manufacturer’s guidelines. Random primers were used for cDNA generation. Subsequently, the cDNA samples were adjusted to a concentration of 10 ng × µL^−1^ and stored at −20 °C. A pair of primers (forward and reverse) targeting the *OsCPK5* gene was designed at an exon-exon junction to increase the amplification specificity of the transcribed gene sequence. The OligoPerfect™ Designer program (Invitrogen, Carlsbad, CA, USA, https://www.thermofisher.com/br/en/home/life-science/oligonucleotides-primers-probes-genes/custom-dna-oligos/oligo-design-tools.html (accessed on 26 March 2019) was used for primer design.

Primer concentrations for the reference genes and the *OsCPK5* gene were adjusted for the sample set. Amplification efficiencies of these genes were determined from the slope in efficiency curves on CT plots against the logarithm base two of initial template concentrations. The efficiency (E) values were calculated using the equation [E = 10(−1/slope − 1) × 100] [61]. The correlation coefficient values (r^2^) were also determined and signified the linearity of the standard curve, where values near 1 are ideal, as are values of the standard curve’s slope, which should approximately be −3.32, indicating a PCR with 100% efficiency. Each reaction had a final volume of 10.0 μL, comprising 5 ng of cDNA, a pair of primers (forward/reverse) with adjusted concentrations for the sample, and 5 μL of PowerUp™ SYBR^®^ Green Master Mix (Thermo Fisher, Waltham, MA, USA). The amplification conditions were as follows: 50 °C for 2 min (UDG incubation); 95 °C for 2 min (cDNA denaturation); and 40 cycles of 95 °C for 15 s and 60 °C for 30 s (annealing and extension). RT-qPCRs were conducted and analyzed on a 7500 Real Time PCR System (Applied Biosystems^®^, Waltham, MA, USA). The stability of the reference genes actin (*ACT*) and eukaryotic elongation factor-1α (*eEF-1α*), used for data normalization, was determined using NormFinder version 20 software [62].

In gene expression analysis, the comparative CT method (ΔΔCT) was used to determine the relative quantification (RQ) with the DataAssist program version 3.01 (Life Technologies). Relative quantification of the *OsCPK5* gene was conducted with three technical replicates comparing the normalized target quantity in each sample (within its respective treatment) to the normalized target quantity in the BRSMG Curinga control plants (NGM). The ΔCt value for each sample was obtained by subtracting the Ct values of the reference genes from the Ct values of the gene of interest. The ΔΔCt value was calculated using the following formula: ΔΔCt = ΔCt (sample) − ΔCt (normalizing sample). Subsequently, the formula 2^(−ΔΔCt)^ was applied to obtain the relative expression value of the gene under investigation. The normalized Ct values of the target gene were subjected to ANOVA and Tukey’s test (*p* < 0.05) using R software version 3.5.3.

### 4.5. RNA-Seq and Bioinformatics Analysis

For transcriptome analysis, eight leaf tissue samples were collected from the GM and NGM plants (two biological replicates for each irrigation treatment) at stage R6. RNA extraction was carried out using a PureLink^®^ RNA Mini Kit (Ambion^®^, Carlsbad, CA, USA) following the manufacturer’s protocol. The RNA quantity and quality were assessed using a Qubit^®^ 2.0 fluorometer and a BioAnalyzer 2100), respectively. Transcriptome sequencing (RNA-seq) was performed on an Illumina HiSeq 2000 platform (Genone Ltd., Rio de Janeiro, Brazil). The raw data in fastq were initially processed by removing adapter-containing and low-quality reads. The reference genome and gene model annotation files were directly sourced from the genome website (*Oryza sativa* ssp. Japonica, Nipponbare—MSU Rice Genome version 7.0). An index of the reference genome was constructed, and paired-end clean reads were aligned using HISAT2 v2.0.5 [63].

Mapped reads from each sample were assembled using StringTie v1.3.3b [64]. The gene expression levels were quantified using FeatureCounts v1.5.0-p3 [65], which counts the mapped reads per gene. Subsequently, the FPKM (fragments per kilobase of transcript sequence per million base pairs sequenced) of each gene was calculated based on gene length and the mapped read count. Differential expression analysis, involving two conditions/groups (with two biological replicates per condition), was conducted using the DESeq2R package version 1.20.0 [66]. Genes with an adjusted *p*-value of ≤ 0.05 were identified as differentially expressed. Gene ontology (GO) enrichment analysis of these differentially expressed genes was performed using the clusterProfiler version 4.0 R package with gene length bias correction [67]. GO terms with a corrected *p*-value of ≤ 0.05 were considered significantly enriched. Further GO enrichment analysis was carried out using the singular enrichment analysis tool (SEA, http://www.broadinstitute.org/gsea/index.jsp (accessed on 4 April 2023)) with a false discovery rate (FDR) and *p*-value ≤ 0.05. Additionally, differentially expressed genes were placed within metabolic pathways utilizing the Kyoto Encyclopedia of Genes and Genomes (KEGG), available at http://www.genome.jp/kegg/ (accessed on 18 April 2023).

## 5. Conclusions

In conclusion, *OsCPK5* overexpression induced diverse drought tolerance mechanisms, indicating the potential for future development of more drought-tolerant rice cultivars.

## Figures and Tables

**Table 1:** Average agronomic performance of *OsCPK5*_E4 (GM) and BRSMG Curinga (NGM) rice plants: grain yield, number of filled grains, harvest index, tiller number, panicle number, flag leaf length and width, and dry and fresh mass under control and water deficit (WD) irrigation treatments.

Trait	Genotype	Control	WD
Grain yield (g plant^−1^)	GM	42.7 ± 6.3 ^Aa^	20.2 ± 4.2 ^Ab^
	NGM	43.5 ± 9.9 ^Aa^	19.5 ± 4.6 ^Ab^
Number of filled grains	GM	357 ± 23.6 ^Aa^	155 ± 35.7 ^Ab^
	NGM	338 ± 93.1 ^Aa^	186 ± 37.3 ^Ab^
Number of empty grains	GM	51.8 ± 7.7 ^Ab^	159.8 ± 64 ^Aa^
	NGM	82.3 ± 12.2 ^Ab^	192.3 ± 22.4 ^Aa^
Harvest index	GM	46.9 ± 2 ^Aa^	26.5 ± 3.8 ^Ab^
	NGM	41.7 ± 2.6 ^Ba^	25.8 ± 6 ^Ab^
Tiller number	GM	20.7 ± 3.2 ^Aa^	22.6 ± 2.4 ^Aa^
	NGM	23.4 ± 1.7 ^Aa^	22.8 ± 4.9 ^Aa^
Panicle number	GM	20.7 ± 3.2 ^Aa^	22.5 ± 2.6 ^Aa^
	NGM	23.4 ± 1.7 ^Aa^	22.2 ± 4.9 ^Aa^
Flag leaf length (cm)	GM	18.6 ± 2.7 ^Aa^	17.4 ± 2.2 ^Aa^
	NGM	17.8 ± 1.1 ^Aa^	18.5 ± 2 ^Aa^
Flag leaf width (mm)	GM	16.2 ± 0.8 ^Aa^	15.4 ± 1.5 ^Aa^
	NGM	15.5 ± 0.6 ^Aa^	14.7 ± 0.8 ^Aa^
Dry mass	GM	48.6 ± 8.1 ^Aa^	58.6 ± 10.9 ^Aa^
	NGM	59.3 ± 6.8 ^Aa^	57.8 ± 13.2 ^Aa^
Fresh mass	GM	185.4 ± 23.4 ^Aa^	131.6 ± 26.7 ^Ab^
	NGM	169.7 ± 24 ^Aa^	158.3 ± 38.4 ^Aa^

Uppercase letters indicate a comparison between GM (genetically modified) and NGM (not genetically modified) plants for the same treatment (columns), while lowercase letters indicate a comparison of water treatments for the same genotype (lines) (Tukey’s HSD test at 5% probability and n = 4). Data are expressed as mean ± standard deviation.

**Table 2:** Values of photosynthetic rate (A), stomatal conductance (gs), instantaneous water-use efficiency (iWUE), intrinsic water-use efficiency (WUEintr), transpiration rate (E), internal carbon concentration (Ci), and carboxylation efficiency (A/Ci) in the NGM plants (wild-type) and GM plants (overexpressing the *OsCPK5* gene) throughout stages R3 (79 DAS—beginning of water deficit), R6 (93 DAS—end of water deficit), and R8 (101 DAS—7 days after return to regular irrigation).

Genotype	Stage R3 (79 DAS)		Stage R6 (93 DAS)		Stage R8 (101 DAS)	
	Control	WD	Control	WD	Control	WD
A (GM)	8.25 ± 1.9 ^Aa^	8.18 ± 1.7 ^Aa^	6.71 ± 2.27 ^Aa^	4.37 ± 1.5 ^Ab^	7.9 ± 1.44 ^Aa^	7.07 ± 2.76 ^Aab^
A (NGM)	9.99 ± 2.1 ^Aa^	9.85 ± 1.97 ^Aa^	4.83 ± 2.83 ^Ab^	5.96 ± 2.14 ^Ab^	10.2 ± 2.17 ^Aa^	5.59 ± 2.08 ^Ab^
gs (GM)	0.19 ± 0.03 ^Aa^	0.16 ± 0.02 ^Aa^	0.08 ± 0.02 ^Aa^	0.04 ± 0.03 ^Bb^	0.21 ± 0.02 ^Aa^	0.15 ± 0.02 ^Ab^
gs (NGM)	0.18 ± 0.02 ^Aa^	0.16 ± 0.01 ^Aa^	0.11 ± 0.03 ^Aa^	0.08 ± 0.02 ^Ab,^*	0.21 ± 0.02 ^Aa^	0.18 ± 0.02 ^Aa^
iWUE (GM)	2.2 ± 0.4 ^Aa^	2.2 ± 0.54 ^Aa^	2.6 ± 0.89 ^Aa^	3.1 ± 0.96 ^Aa^	2.1 ± 0.47 ^Aa^	2.4 ± 0.94 ^Aa^
iWUE (NGM)	2.5 ± 0.62 ^Aab^	2.6 ± 0.49 ^Aa^	1.5 ± 1.11 ^Bb,^*	2.4 ± 0.19 ^Aa^	2.9 ± 0.46 ^Aa^	1.7 ± 0.56 ^Ba^
WUEintr (GM)	44.6 ± 8.3 ^Ab^	50.5 ± 7.1 ^Ab^	81.8 ± 18.0 ^Aa^	109.2 ± 40.57 ^Aa^	38.1 ± 10.32 ^Ab^	48.1 ± 24.62 ^Ab^
WUEintr (NGM)	57.1 ± 14.5 ^Aa^	62.7 ± 9.53 ^Aab^	43.1 ± 15.5 ^Ba,^*	79.5 ± 7.1 ^Aa^	49.7 ± 8.91 ^Aa^	31.1 ± 13.82 ^Ab^
E (GM)	3.76 ± 0.7 ^Aa^	3.68 ± 0.57 ^Aa^	2.56 ± 0.89 ^Aa^	1.42 ± 0.71 ^Bb^	3.71 ± 0.35 ^Aa^	3.00 ± 0.15 ^Aa^
E (NGM)	3.94 ± 0.61 ^Aa^	3.83 ± 0.37 ^Aa^	3.19 ± 0.5 ^Aa^	2.45 ± 0.89 ^Aa,^*	3.52 ± 0.39 ^Aa^	3.27 ± 0.13 ^Aa^
Ci (GM)	300.5 ± 29 ^Aa^	279.8 ± 14.7 ^Aa^	274.0 ± 25.94 ^Aa^	236.0 ± 49.74 ^Aa^	291.0 ± 64.71 ^Aa^	278.0 ± 53.03 ^Aa^
Ci (NGM)	274.0 ± 25 ^Aa^	260.3 ± 25.1 ^Aa^	291.0 ± 64.71 ^Aa^	245.5 ± 27.3 ^Aa^	284.5 ± 22.55 ^Aa^	290.0 ± 16.79 ^Aa^
A/Ci (GM)	0.03 ± 0.01 ^Aa^	0.03 ± 0.01 ^Aa^	0.02 ± 0.01 ^Aa^	0.02 ± 0.01 ^Aa^	0.03 ± 0.01 ^Aa^	0.03 ± 0.02 ^Aa^
A/Ci (NGM)	0.04 ± 0.01 ^Aa^	0.04 ± 0.01 ^Aa^	0.02 ± 0.01 ^Aa^	0.02 ± 0.01 ^Aa^	0.04 ± 0.01 ^Aa^	0.02 ± 0.01 ^Ab^

* Significant differences between the GM and NGM genotypes under the two irrigation conditions and collection periods separately. The capital letter indicates a significant difference between plants of the same genotype under different irrigation conditions in each collection period. The lowercase letter indicates a significant difference between plants of the same genotype and under the same irrigation conditions in the three collection periods (Tukey’s test, *p* < 0.05, and n = 4). Data are shown as mean ± standard deviation.

**Table 3:** Chlorophyll a/b ratio in the NGM plants (wild-type) and GM plants (overexpressing the *OsCPK5* gene) throughout stages R3 (79 DAS—before cutting irrigation), R6 (93 DAS—after cutting irrigation), and R8 (101 DAS—7 days after returning to normal irrigation). WD: water deficit.

Genotype	Stage R3 (79 DAS)			Stage R6 (93 DAS)			Stage R8 (101 DAS)		
	Control	WD	r. (%)	Control	WD	r. (%)	Control	WD	r. (%)
GM	2.4 ± 0.29 ^Aa^	2.6 ± 0.17 ^Aa^	-	2.7 ± 0.33 ^Aa^	2.4 ± 0.35 ^Aa^	8.4	2.8 ± 0.1 ^Aa^	2.5 ± 0.17 ^Aa^	8.1
NGM	1.8 ± 0.7 ^Ab,^*	2.0 ± 0.93 ^Ab,^*	-	2.9 ± 0.22 ^Aa^	2.4 ± 0.37 ^Aa^	14.8	2.5 ± 0.1 ^Ab^	2.2 ± 0.18 ^Bb^	13.7
Variation (%)	25	23.1					10.7	12	

The capital letter indicates a significant difference between plants of the same genotype under different irrigation conditions in each collection period. The lowercase letter indicates a significant difference between plants of different genotypes under the same irrigation conditions. * Significant differences between the GM and NGM genotypes under the same irrigation conditions and collection time. r. (%) indicates the percentage decrease in the proportions of chlorophyll a and b in plants of the same genotype under water deficit compared to the control (Tukey’s test, *p* < 0.05, and n = 4). Data are expressed as mean ± standard deviation.

**Table 4:** Description of the sequenced libraries (RNA-seq) derived from leaf tissues in the GM and NGM genotypes.

Library	Raw Reads Avg.	Clean Reads Avg.	Reads Aligned Avg.	Reads Aligned to Exons
GM drought	46,065,473	45,329,575	43,432,707 (95.8%)	5,841,166,813 (89.9%)
GM control	43,492,722	42,764,401	40,969,707 (95.8%)	5,473,640,936 (89.3%)
NGM drought	43,542,120	42,639,242	40,547,019 (95.1%)	5,422,323,506 (89.3%)
NGM control	44,357,608	43,611,743	41,666,443 (95.5%)	5,537,551,774 (88.8%)
Average	44,364,481	43,586,240	41,653,969 (95.6%)	5,568,670,757 (89.3%)

**Table 5:** Determination of the average numbers of the most abundant RNA-seq transcripts.

Gene ID	GM Drought	GM Control	NGM Drought	NGM Control	Total	Description ^(1)^
Os11g0707000	861,550	861,372	783,893.5	709,240	3,216,055.5	Rubisco activase
Os11g0171300	415,106	403,158.5	388,764.5	284,405.5	1,491,434.5	Chloroplast aldolase
Os12g0190000	68,818	137,986	130,697.5	120,118	457,619.5	GDP-L-galactose phosphorylase
Os08g0157600	85,963	86,221.5	100,490	107,916.5	380,591	Circadian clock associated 1
Os09g0346500	114,615.5	147,237.5	75,065.5	37,457	374,375.5	Chlorophyll a/b-binding protein
Os01g0200700	136,507	101,727.5	66,638.5	58,841	363,714	Metallothionein i-3a
Os08g0139700	15,126	31,237.5	140,959.5	161,352	348,675	Terpene synthase 29
Os08g0200300	107,531	94,602	77,955.5	53,800.5	333,889	Photosystem II subunit PsbR3
Os05g0202800	95,020.5	106,567.5	40,654.5	66,918.5	309,161	Metallothionein 3b
Os07g0637300	58,555	53,547	70,199.5	73,397	255,698.5	Pyruvate dehydrogenase kinase
Os02g0685900 *	2.5	2.5	1.5	2	8.5	Calcium-dependent protein kinase 5

* Transcript of the *OsCPK5* gene, overexpressed in GM plants, is presented in the table for comparison. Overall, it was the 22,850th most expressed transcript. ^(1)^ According to the Rice Annotation Project (RAP).

**Table 6:** Differentially expressed genes upregulated in the “response to osmotic stress” class, category BP, in rice plants genetically modified with the *OsCPK5* gene (GM) compared to the non-genetically modified BRSMG Curinga (NGM). The Log2 values are related to the gene expression differences between the GM and NGM plants.

Gene ID	Description ^(1)^	Log2	*p*-Value
Os01g0136200	Class I heat shock protein 2 (16.9 kDa)	3.05 × 10^14^	1.78 × 10^10^
Os03g0266300	Class I heat shock protein 1 (17.9 kDa)	1.79 × 10^14^	1.83 × 10^−2^
Os03g0626500	Unknown	1.70 × 10^14^	3.93 × 10^−1^
Os01g0136100	Class I heat shock protein 1 (16.9 kDa)	3.50 × 10^14^	1.96 × 10^3^
Os09g0110300	Cyclase-like 4	1.75 × 10^14^	7.67 × 10^5^
Os03g0643300	Ornithine aminotransferase	1.34 × 10^14^	1.07 × 10^7^
Os01g0667200	Glyoxalase II-1	1.59 × 10^14^	1.72 × 10^7^
Os10g0471100	Wax-deficient anther 1	2.40 × 10^14^	2.40 × 10^9^
Os03g0267000	Class I heat shock protein (18.0 kDa)	1.58 × 10^14^	0.00250
Os03g0281900	ABC transporter G family member 5	2.12 × 10^14^	0.00446
Os07g0517100	HSP18.8	1.32 × 10^14^	0.00507

^(1)^ According to the Rice Annotation Project (RAP).

**Table 7:** Differentially expressed genes downregulated in the “regulation of response to stress” class, category BP, in rice plants genetically modified with the *OsCPK5* gene (GM) compared to the non-genetically modified BRSMG Curinga (NGM). The Log2 values are related to the gene expression differences between the GM and NGM plants.

Gene ID	Description ^(1)^	Log2	*p*-Value
Os09g0417800	*WRKY* gene 62	−1.06 × 10^14^	1.91 × 10^−44^
Os03g0402800	*TIFY* gene 10A	−1.94 × 10^14^	7.15 × 10^−31^
Os09g0439200	*TIFY* gene 10C	−3.36 × 10^14^	1.56 × 10^−24^
Os10g0392400	*TIFY* gene 11D	−3.13 × 10^14^	6.93 × 10^−23^
Os01g0130200	NRR	−2.27 × 10^14^	9.62 × 10^−16^
Os07g0615200	*TIFY* gene 10B	−1.61 × 10^14^	1.44 × 10^−15^
Os03g0180900	*TIFY* gene 11C	−2.50 × 10^14^	6.10 × 10^−13^
Os01g0508500	NRR repressor homologue 2	−3.57 × 10^14^	5.80 × 10^−8^
Os03g0181100	*TIFY* gene 11B	−2.14 × 10^14^	8.51 × 10^−5^
Os11g0195500	Phytoalexin deficient 4	−1.21 × 10^14^	4.10 × 10^−2^
Os01g0194300	NPR1 homolog 1	−1.50 × 10^14^	2.21 × 10^3^
Os01g0221100	Jasmonyl-L-isoleucine synthase 2	−2.67 × 10^13^	4.40 × 10^6^
Os04g0395800	*TIFY* GENE 9	−4.43 × 10^14^	6.34 × 10^7^
Os06g0603600	*Oryza sativa* SYG/PHO8/XPR1 (SPX) domain gene	−1.15 × 10^14^	2.03 × 10^9^
Os03g0180800	*TIFY* gene 11A	−2.94 × 10^14^	4.14 × 10^9^
Os12g0197500	Suppressor of gene silencing 3	−1.34 × 10^14^	4.18 × 10^9^
Os03g0285800	Multiple stress responsive Map kinase 2	−1.08 × 10^14^	0.00014
Os01g0508100	NRR repressor homologue 3	−6.61 × 10^14^	0.000275
Os05g0368100	Unknown	−2.87 × 10^14^	0.000859
Os10g0391400	*TIFY* gene 11E	−2.01 × 10^14^	0.000886

^(1)^ According to the Rice Annotation Project (RAP).

**Table 8:** Differentially expressed genes associated with two drought-related KEGG metabolic pathways. The Log2 values are related to the gene expression differences between the GM and NGM plants.

Pathway	Gene ID	Description ^(1)^	Log2
Plant hormone signal transduction (osa040705); *p*-value: 0.003	Upregulated		
	Os02g0796500	B-Type response regulator 3	1.04 × 10^14^
	Os10g0564500	Stress/ABA-activated protein kinase 3	1.03 × 10^14^
	Os08g0176900	b-ZIP transcription factor 64	1.33 × 10^14^
	Os03g0297600	Regulatory components of ABA receptor 4	1.49 × 10^14^
	Downregulated		
	Os03g0402800	*TIFY* gene 10A	−1.94 × 10^14^
	Os09g0439200	*TIFY* gene 10C	−3.36 × 10^14^
	Os10g0392400	*TIFY* gene 11D	−3.13 × 10^14^
	Os07g0615200	*TIFY* gene 10B	−1.61 × 10^14^
	Os03g0180900	*TIFY* gene 11C	−2.50 × 10^14^
	Os03g0667100	NPR1-like gene 3	−2.94 × 10^14^
	Os03g0181100	TIFY gene 11B	−2.14 × 10^14^
	Os11g0143300	A-Type response regulator 9	−1.24 × 10^14^
	Os12g0139400	A-Type response regulator 10	−1.12 × 10^14^
	Os01g0194300	NPR1 homolog 1	−1.50 × 10^14^
	Os01g0221100	Jasmonyl-L-isoleucine synthase 2	−2.67 × 10^13^
	Os04g0395800	*TIFY* gene 9	−4.43 × 10^14^
	Os04g0673300	A-Type response regulator 6	−1.10 × 10^14^
	Os01g0382000	Pathogenesis-related gene 1B	−7.47 × 10^14^
	Os03g0180800	*TIFY* gene 11A	−2.94 × 10^14^
	Os10g0391400	*TIFY* gene 11E	−2.01 × 10^14^
	Os01g0221000	Unknown	−1.89 × 10^14^
MAPK signaling pathway (osa04016); *p*-value: 0.04	Upregulated		
	Os10g0564500	Stress/ABA-activated protein kinase 3	1.03 × 10^14^
	Os03g0297600	Regulatory components of ABA receptor 4	1.49 × 10^14^
	Os04g0556000	Heavy metal ATPase 5	1.09 × 10^14^
	Downregulated		
	Os03g0132900	Chitinase 11	−3.57 × 10^13^
	Os06g0726200	Chitinase 1	−2.37 × 10^14^
	Os09g0438000	Respiratory burst oxidase homolog G	−1.74 × 10^14^
	Os03g0743500	Calmodulin-like protein 4	−1.60 × 10^14^
	Os10g0542900	Chitinase 8	−2.63 × 10^14^
	Os01g0382000	Pathogenesis-related gene 1B	−7.47 × 10^14^
	Os04g0578000	ACC synthase 2	−3.98 × 10^14^
	Os03g0285800	Multiple stress responsive map kinase 2	−1.08 × 10^14^
	Os05g0474800	*WRKY* gene 70	−1.30 × 10^14^

^(1)^ According to the Rice Annotation Project (RAP).

## Data Availability

Data are contained within the article and Supplementary Materials. The datasets supporting the findings of this article are included within the article. The data are available from the corresponding author on reasonable request.

## Supplementary Materials

The following supporting information can be downloaded at: https://www.mdpi.com/article/10.3390/plants12223826/s1.
