# Supplementary material for: Calcium-Dependent Protein Kinase 5 (OsCPK5) Overexpression in Upland Rice (Oryza sativa L.) under Water Deficit
Source: Plants (Basel). 2023 Nov 11;12(22):3826. doi: 10.3390/plants12223826 (PMC10674721; doi:10.3390/plants12223826)
Supplement: Supplementary file 1 [file plants-12-03826-s001.zip › plants-2603682-supplementary.pdf]

Table S1. Average agronomic performance of *OsCPK5*_E4 (GM) and BRSMG Curinga (NGM) rice plants: grain yield, number of filled grains, harvest index, tiller number, panicle number, flag leaf length and width, dry and fresh mass, in control and water deficit (WD) irrigation treatments.

**Trait**	**Genotype**	**Control**	**WD**
Grain yield (g plant^{-1})	*OSCPK5_E2*	73.8 (18.6) A	12.3 (10.2) B
	OSCPK5_E4	85.5 (20.3) A	30.9 (20.5) A
	OSCPK5_E7	74.8 (16.0) A	9.12 (10.9) B
	NGM	87.1 (20.1) A	29.5 (21.0) A
Percentage of filled grains	*OSCPK5_E2*	88.8 (0.02) A	16.8 (0.1) B
	OSCPK5_E4	88.3 (0.04) A	38.3 (0.2) A
	OSCPK5_E7	87.5 (0.06) A	17.8 (0.1) B
	NGM	79.8 (0.05) A	37.3 (0.2) A
Plant Height	*OSCPK5_E2*	105.4 (8.2) A	94.6 (8.8) C
	OSCPK5_E4	90.1 (7.2) A	81.4 (3.2) A
	OSCPK5_E7	102.8 (8.6) A	87.9 (6.0) B
	NGM	105.6 (11.5) A	94.5 (5.1) C
Tiller Number	*OSCPK5_E2*	40.3 (5.6) A	42.5 (7.2) A
	OSCPK5_E4	41.5 (9.3) A	45.3 (4.2) A
	OSCPK5_E7	36.3 (3.5) A	44.0 (7.4) A
	NGM	46.8 (2.5) A	45.8 (7.1) A
Panicle Number	*OSCPK5_E2*	40.3 (5.9) A	41.3 (7.2) A
	OSCPK5_E4	41.5 (9.3) A	43.8 (4.2) A
	OSCPK5_E7	36.3 (3.5) A	44.5 (7.1) A
	NGM	46.8 (2.5) A	45.0 (7.8) A
Flag leaf width (cm)	*OSCPK5_E2*	1.48 (0.2) A	1.51 (0.1) A
	OSCPK5_E4	1.62 (0.1) A	1.54 (0.2) A
	OSCPK5_E7	1.58 (0.1) A	1.58 (0.1) A
	NGM	1.51 (0.1) A	1.50 (0.1) A
Flag leaf length (mm)	*OSCPK5_E2*	19.0 (3.5) A	17.4 (2.1) A

OSCPK5_E4	18.6 (2.7) A	17.4 (2.3) A
OSCPK5_E7	18.6 (1.5) A	17.4 (2.1) A
NGM	17.1 (1.5) A	19.5 (3.6) A

Uppercase letters indicate a comparison between *OSCPK5_E4* (genetically modified) and *OSCPK5_E4* (not genetically modified) plants for the same treatment (Scott-Knott test at 5% probability and n = 4). In parentheses: Standard deviation values.
